# Metabolic engineering of *Escherichia coli* for production of 2‐Phenylethylacetate from L‐phenylalanine

**DOI:** 10.1002/mbo3.486

**Published:** 2017-04-24

**Authors:** Daoyi Guo, Lihua Zhang, Hong Pan, Xun Li

**Affiliations:** ^1^ College of Life and Environmental Sciences Gannan Normal University Ganzhou China; ^2^ Key Laboratory of Organo‐Pharmaceutical Chemistry Gannan Normal University Ganzhou Jiangxi Province China

**Keywords:** 2‐Phenylethanol, 2‐Phenylethylacetate, *Escherichia col*i, Metabolic engineering

## Abstract

In order to meet the need of consumer preferences for natural flavor compounds, microbial synthesis method has become a very attractive alternative to the chemical production. The 2‐phenylethanol (2‐PE) and its ester 2‐phenylethylacetate (2‐PEAc) are two extremely important flavor compounds with a rose‐like odor. In recent years, *Escherichia coli* and yeast have been metabolically engineered to produce 2‐PE. However, a metabolic engineering approach for 2‐PEAc production is rare. Here, we designed and expressed a 2‐PEAc biosynthetic pathway in *E. coli*. This pathway comprised four steps: aminotransferase (ARO8) for transamination of L‐phenylalanine to phenylpyruvate, 2‐keto acid decarboxylase KDC for the decarboxylation of the phenylpyruvate to phenylacetaldehyde, aldehyde reductase YjgB for the reduction of phenylacetaldehyde to 2‐PE, alcohol acetyltransferase ATF1 for the esterification of 2‐PE to 2‐PEAc. Using the engineered *E. coli* strain for shake flasks cultivation with 1 g/L L‐phenylalanine, we achieved co‐production of 268 mg/L 2‐PEAc and 277 mg/L 2‐PE. Our results suggest that approximately 65% of L‐phenylalanine was utilized toward 2‐PEAc and 2‐PE biosynthesis and thus demonstrate potential industrial applicability of this microbial platform.

## 1 Introduction

2‐phenylethylacetate (2‐PEAc), as a high‐value aromatic ester with a rose‐like odor, is in high demand and widely used as an additive in food, drinks, perfumes, cosmetics, and household products (Kuo, Chen, Chen, Liu, & Shieh, 2014). In addition, due to its high‐energy density, 2‐PEAc is a potential fuel molecule. Currently, 2‐PEAc is mainly produced by chemical synthesis (Manabe, Iimura, Sun, & Kobayashi, 2002). Although the chemically synthesized 2‐PEAc has strong advantages in cost and yield, consumers prefer more natural flavor compounds in the field of food and cosmetics (Etschmann, Bluemke, Sell, & Schrader, 2002). However, production of natural 2‐PEAc by extraction from the essential oils of flowers and plants is costly and labor‐intensive (Etschmann et al., 2002). Therefore, the production of 2‐PEAc using engineered microorganisms can be an attractive alternative to the traditional low‐yielding and costly extraction and distillation processes.

**Figure 1:**
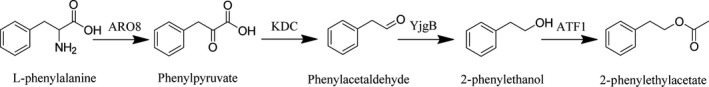
Engineered pathways for production of 2‐PEAc from L‐phenylalanine. Aminotransferase ARO8 for transamination of L‐phenylalanine to phenylpyruvate, 2‐keto acid decarboxylase KDC for the decarboxylation of the phenylpyruvate to phenylacetaldehyde and aldehyde reductase YjgB for the reduction of phenylacetaldehyde to 2‐PE. 2‐PEAc were produced from 2‐PE and acetyl‐CoA through the expression of alcohol acetyltransferase ATF1 that catalyzes the esterification of 2‐PE and acetyl‐CoA

Many yeast strains naturally produce small amounts 2‐phenylethanol (2‐PE) and 2‐PEAc from the catabolism of amino acids via the Ehrlich pathway: phenylpyruvate is decarboxylated to phenylacetaldehyde, then reduced to 2‐PE, and finally, esterified to 2‐PEAc (Etschmann & Schrader, 2006). Several wild yeast have been evaluated the capacity of synthetic 2‐PEAc (Adler, Hugen, Wiewiora, & Kunz, 2011; Akita, Ida, Obata, & Hara, 1990; Lilly, Lambrechts, & Pretorius, 2000; Viana, Belloch, Vallés, & Manzanares, 2011; Viana, Gil, Vallés, & Manzanares, 2009; Wittmann, Hans, & Bluemke, 2002). Recently, microbial production of 2‐PE in engineered *E. coli* or yeast was reported (Kim, Cho, & Hahn, 2014; Kim, Lee, & Oh, 2014; Yigal, Zohar, & Ayelet, 2014; Yin et al., 2015). However, the production of 2‐PEAc by engineered micro‐organisms is rare. In this study, we engineered *E. coli* to produce 2‐PE by overexpressing 2‐keto acid decarboxylase (KDC), and aldehyde reductase (YigB). Then we further generated a 2‐PEAc biosynthetic pathway in this 2‐PE‐producing strain by overexpressing an alcohol acetyltransferase (ATF1) for the subsequent esterification 2‐PE to 2‐PEAc. Finally, we improved 2‐PEAc production by increasing the precursor substrate availability (Figure 1).

## 2 Materials and Methods

### 2.1 Enzymes and DNA kits

Phusionpolymerase and T4 ligase were purchased from New England Biolabs. Plasmid mini kits, PCR purification kits and gel extraction kits were ordered from Fermentas (Burlington, Canada).

### 2.2 Plasmid construction for 2‐PEAc production

The KDC (GenBank NP_010668.3), ADH6 (GenBank AJS99695.1), and ATF1 (GenBank NP_015022.3) gene were individually amplified by PCR from *Saccharomyces cerevisiae* YPH499 (Invitrogen) genomic DNA using primers KDC‐*XbaI*/KDC‐*NheI*‐*BamHI*, ADH6‐*XbaI*/ADH6‐*NheI‐BamHI*, and ATF1‐*XbaI*/ATF1‐*NheI‐SacI* separately, and individually inserted into pET28a(+) to give pPG30, pPG31 and pPG32. YjgB (GenBank CAQ34616.1) gene from *E. coli* was amplified by PCR using primers YjgB‐*XbaI*, and YjgB‐*NheI*‐*BamHI*, and inserted into pET28a(+) to give pDG33. The *XbaI*‐*XhoI* fragment of YjgB from pPG32 was inserted into *NheI* and *XhoI* sites of pPG30 to give pPG34. The *XbaI*‐*XhoI* fragment of ADH6 from pPG31 was inserted into *NheI* and *XhoI* sites of pPG30 to give pPG35. The *XbaI*‐*XhoI* fragment of ATF1 from pPG33 was inserted into *NheI* and *XhoI* sites of pPG34 to give pPG36. ARO8 (GenBank EWH18548.1) gene from *S. cerevisiae* YPH499 was amplified by PCR using primers ARO8‐*XbaI*, and ARO8‐*XhoI*, and inserted into *NheI* and *XhoI* sites of pPG36 to give pPG37. The sequences of all primers used in PCRs are listed in Table 1. Plasmids used in this study are listed in Table 2.

**Table 1:** Primers used in this study

Primer name	Sequence (5′‐3′)
KDC‐ *XbaI*	ATCTCTAGATTTAAGAAGGAGATATAATGGCACCTGTTACAATTGAAAAGTTC
KDC‐ *NheI*‐*BamHI*	TCTGGATCCGCTAGCCTATTTTTTATTTCTTTTAAGTGCCGCTG
ADH6‐*XbaI*	TTGTCTAGATTTAAGAAGGAGATATAATGTCTTATCCTGAGAAATTTGAAGGTATCG
ADH6‐*NheI*‐*BamHI*	TCTGGATCCGCTAGCCTAGTCTGAAAATTCTTTGTCGTAGCCGA
YjgB‐*XbaI*	ATATCTAGATTTAAGAAGGAGATATAATGTCGATGATAAAAAGCTATGCCG
YjgB‐*NheI*‐*BamHI*	TCTGGATCCGCTAGCTCAAAAATCGGCTTTCAACACCAC
ATF1‐*XbaI*	GGATCTAGAAACTTTAAGAAGGAGATATAATGAATGAAATCGATGAGAAAAATCAGG
ATF1‐*NheI*‐*SacI*	GATGAGCTCGCTAGCCTAAGGGCCTAAAAGGAGAGCTTTGTAA
ARO8‐*XbaI*	AACTCTAGATTTAAGAAGGAGATATAATGATGACTTTACCTGAATCAAAAGACTTTTC
ARO8‐*XhoI*	ACACTCGAGCTATTTGGAAATACCAAATTCTTCGTATAA

**Table 2:** Plasmids used in this study

Plasmids	Replication origin	Overexpressed genes	Resistance	Source
pPG30	pBR322	P_T7_: *kdc*	Kan	This study
pPG31	pBR322	P_T7_: *adh6*	Kan	This study
pPG32	pBR322	P_T7_: *atf1*	Kan	This study
pPG33	pBR322	P_T7_:*yjgB*	Kan	This study
pPG34	pBR322	P_T7_: *kdc* and *yjgB*	Kan	This study
pPG35	pBR322	P_T7_: *kdc* and *adh6*	Kan	This study
pPG36	pBR322	P_T7_: *kdc*,* yjgB* and *atf1*	Kan	This study
pPG37	pBR322	P_T7_: *kdc*,* yjgB, atf1*and *aro8*	Kan	This study

### 2.3 Cell transformation

*Escherichia coli* strain MG1655 competent cells were transformed using plasmids pPG30‐37. Cells were selected on LB plates containing kanamycin (50 mg/L).

### 2.4 Shake flask cultures

Recombinant strains of *E. coli* were streaked onto LB agar plates with antibiotics (50 mg/L kanamycin) and incubated at 37°C for 12–20 hr. Single colonies were picked and inoculated into 5 ml LB medium in 20‐ml flasks. Flasks were incubated at 37°C in a rotary shaker at 200 rpm for 12 hr. Cells were collected by centrifugation at 4,000 g for 1 min, resuspended in 100 ml modified M9 medium as previously described by Guo, Zhu, Deng, & Liu (2014), and shaken as above. When the OD_600_ reached 0.8, IPTG was added to a final concentration of 0.1 mmol/L. Samples were taken at 28 hr after fermentation and analyzed by GC/MS.

### 2.5 GC/MS analysis of 2‐PE and 2‐PEAc

Cultures samples (15 ml) were transferred to 50‐ml glass tubes with glass stop corks. Glass beads were added to disrupt the cells by vigorous shaking. 2‐PE or 2‐PEAc was extracted using 15 ml chloroform followed by centrifugation at 8,000*g* for 3 min. The chloroform phase was withdrawn, evaporated to dryness, and redissolved in 0.6 ml chloroform. GC/MS analysis of 2‐PE or 2‐PEAc dissolved in chloroform phase was performed on a Series 7890A GC system equipped with a Series 5975C EI MSD detector (Agilent Technologies, Santa Clara, CA). The chloroform phase (1 μl) was analyzed after splitless injection on a DB‐5 ms capillary column (30 m × 0.25 mm × 0.25 m, Agilent Technologies). Helium was used as the carrier gas. Injector and detector temperatures were 270°C and 250°C, respectively. The following temperature program was applied: 100°C for 3 min, an increase in 12°C/min to 200°C. Quantification was done by using 2‐phenethylpropionat as internal standard.

## 3 Results

### 3.1 Construction of 2‐PE biosynthetic pathway in *Escherichia coli*

The gene KDC which encode 2‐keto acid decarboxylase was amplified from *S. cerevisiae* and introduced to *E. coli* MG1655 strain to yield *E. coli* MG1655/pDG30.

The recombinant and the control strains were cultured in a modified M9 media and the 2‐PE production was analyzed by GC‐MS (Figure S1). No 2‐PE was detected in the negative control strain *E. coli*. In contrast, 85.7 ± 4.34 mg/L of 2‐PE was formed in the recombinant strains (Table 3). This result suggests that there is an endogenous phenylacetaldehyde reductase in *E. coli*.

**Table 3:** 2‐PE and 2‐PEAc production in engineered *E. coli* strains from glucose in shake flasks for 28 hr. All experiments were performed in triplicate and error bars show S.D

Production (mg/L)	The engineered *E. coli* strains			
	MG1655/pDG30^a^	MG1655/pDG34^b^	MG1655/pDG35^c^	MG1655/pDG36^d^
2‐PE	85.7 ± 4.34	180.9 ± 4.23	95.9 ± 5.28	45.9 ± 2.20
2‐PEAc				53.7 ± 2.83

a expression of *kdc*.

b co‐expression of *kdc* and *yjgB*.

c co‐expression of *kdc* and *adh6*.

d co‐expression of *kdc*,* yjgB* and *atf1*.

YjgB has been identified as aldehyde reductase in *E. coli* and can accept a broadly range of various aldehydes as substrates for the various alcohols production (Guo, Hong, & Xun, 2015; Rodriguez & Atsumi, 2014). To improve the production of 2‐PE, YjgB was amplified and co‐expressed with KDC in *E. coli*. The overexpression of the YjgB gene resulted in an approximately 110% increase in 2‐PE production up to 180.9 ± 4.23 mg/L (Table 3). An aldehyde reductase ADH6 gene from *S. cerevisiae*, which shows high activity for the various short‐chain alcohols production (Larroy et al. 2002), was also co‐expressed with KDC and resulted in a small increase of 2‐PE (Table 3). This result suggests that phenylacetaldehyde is not a good substrate for ADH6.

### 3.2 Construction of 2‐PEAc biosynthetic pathway in *Escherichia coli*

Alcohol acyltransferase (AAT) plays a major role in the biosynthesis of acetate esters in plant and yeast. An alcohol acetyltransferase (ATF1) from *S. cerevisiae* was previously discovered and shown to exhibit a broadly substrate specificity for short‐chain alcohols, fatty alcohols and 2‐PE (Guo et al., 2015; Rodriguez, Tashiro, & Atsumi, 2014; Saerens, Delvaux, Verstrepen, & Thevelein, 2010; Tai, Xiong, & Zhang, 2015; Yoshimoto, Fukushige, Yonezawa, & Sone, 2002). After succeeding in demonstrated the biosynthesis of 2‐PE in *E. coli*, we further generated a 2‐PEAc biosynthetic pathway in this 2‐PE‐producing strain by overexpressing ATF1 for the subsequent esterification 2‐PE to 2‐PEAc. 2‐PEAc was extracted from recombinant *E. coli* MG1655/pDG36 and analyzed by GC/MS (Figure S1). Induction of the engineered pathway in recombinant *E. coli* in a modified M9 media resulted in the accumulation of 2‐PEAc with a titer of 53.7 ± 2.83 mg/L (Table 3).

### 3.3 Increase 2‐PEAc production by adding the precursor substrate phenylpyruvate

Phenylpyruvate is the precursor substrate for the biosynthesis of 2‐PEAc. Therefore, we hypothesized that increasing phenylpyruvate availability would improve 2‐PEAc production. In this study, we evaluated the effect of the adding phenylpyruvate (1.0 g/L) for the biosynthesis of 2‐PEAc in engineered strain MG1655/pDG36. Strain grown in 1.0 g/L phenylpyruvate showed a high molar yield. In this medium, MG1655/pDG36 strain produced up to 432.4 ± 8.22 mg/L 2‐PE with the molar yield of 0.58 mol/mol and 247.3 ± 4.99 mg/L 2‐PEAc with the molar yield of 0.25 mol/mol (Figure S2). This results suggested that phenylpyruvate availability is a bottleneck for the biosynthesis of 2‐PEAc.

### 3.4 The biosynthesis of 2‐PEAc from L‐phenylalanine

Although the precursor substrate phenylpyruvate can be de novo synthesized from glucose, the efficiency is quite low because of feedback regulations in many branched metabolic pathways (Manuel et al., 2009). Another strategy is transamination of L‐phenylalanine to phenylpyruvate by aminotransferase. Several groups have demonstrated the biosynthesis of 2‐PE from L‐phenylalanine with a high titer by transamination (Kim, Cho, et al., 2014; Yin et al., 2015).

The gene ARO8 which encode aminotransferase was amplified from *S. cerevisiae* and introduced to 2‐PEAc‐producing strain. In this study, we evaluated the biosynthesis of 2‐PEAc from L‐phenylalanine (the range from 0.5 to 2 g/L) in engineered strain MG1655/pDG37. The titer of 2‐PE and 2‐PEAc is presented in Figure S3. Strain grown in 1.0 g/L of L‐phenylalanine showed the highest the molar yield of 2‐PEAc. In this medium, MG1655/pDG37 strain produced up to 277.6 ± 4.26 mg/L 2‐PE with the molar yield of 0.38 mol/mol and 268.8 ± 4.21 mg/L 2‐PEAc with the molar yield of 0.27 mol/mol (Table 4). A large amount of 2‐PE failed to be effectively converted to 2‐PEAc, suggesting that alcohol acetyltransferase ATF1 is a another bottleneck for the biosynthesis of 2‐PEAc (Figure S3).

**Table 4:** 2‐PE and 2‐PEAc production in engineered strain MG1655/pDG37 (co‐expression of *aro8*,* kdc*,* yjgB* and *atf1*) with modified M9 medium containing 0.5, 1.0, or 2.0 g/L of L‐phenylalanine in shake flasks for 28 hr

Production (mg/L)	The concentration of L‐phenylalanine		
	0.5 g/L	1 g/L	2 g/L
2‐PE	104.1 ± 7.61	277.6 ± 4.26	364.3 ± 9.28
2‐PEAc	199.8 ± 6.55	268.8 ± 4.21	180.7 ± 8.03

All experiments were performed in triplicate and error bars show S.D.

## 4 Discussion

Flavor compounds are in high demand and widely used as an additive in food and cosmetics industry. The production of natural flavor compounds has recently attracted a great deal of interest and represents a challenging target for synthetic biology research (Kuo et al., 2014). In order to meet the need of consumer preferences for natural flavor compounds, microbial synthesis method has become a very attractive alternative to the chemical production.

The 2‐PE and its ester, 2‐PEAc, are two extremely important flavor compounds with a rose‐like odor. In recent years, *E. coli* and yeast have been metabolically engineered to produce 2‐PE, by overexpressing the Ehrlich pathway enzymes 2‐keto acid decarboxylase (KDC) and alcohol dehydrogenase (ADH) (Daisuke, Hayato, Kunihiko, Takashi, & Kiyofumi, 2012; Kim, Cho, et al., 2014). Shen, Nishimura, Matsuda, Ishii, & Kondo, 2016; reported that a 2‐PE biosynthetic *S. cerevisiae* strain with overexpression of ARO10 and deletion of ARO8 (aromatic aminotransferases) produced 96 mg/L 2‐PE (Shen et al., 2016). Kang, Zhang, Du, & Chen, 2014; constructed a heterologous pathway to produce 2‐PE in a phenylalanine‐producing *E. coli*. with overexpression of ADH1 (alcohol dehydrogenase) from *S. cerevisiae* and KDC (phenylpyruvate decarboxylase) from *Pichia pastoris* GS115, this modified strain produced 285 mg/L 2‐PE (Kang et al., 2014). However, a metabolic engineering approach for 2‐PEAc production is rare.

Several enzymatic methods have been reported for the preparation of 2‐PEAc by lipase (Kuo, Liu, Chen, Chang, & Shieh, 2013; Kuo et al., 2012). However, there are some drawbacks by using enzymes in bioprocesses such as the need of long and complicated steps for enzyme isolation and purification. In this study, a fermentative route was created using an engineered *E. coli* for biosynthesis of 2‐PEAc from L‐phenylalanine based on ATF1. The results suggest that approximately 65% of L‐phenylalanine was utilized toward 2‐PEAc and 2‐PE biosynthesis and thus demonstrate potential industrial applicability of this microbial platform.

GC/MS analysis of extracts from the strain of our study revealed that a large number of 2‐PE failed to be effectively converted to 2‐PEAc, suggesting that ATF1 is a bottleneck for further improvement of 2‐PEAc biosynthesis. Thus, improving of the catalytic activity of ATF1 through protein engineering or identification a better alcohol acetyltransferase to replace ATF1 in the future is a promising strategy for enhancing 2‐PEAc production in genetically engineered microorganisms.

## Conflict of Interest

None declared.
