# Supplementary material for: Metabolic engineering of Escherichia coli for production of 2‐Phenylethylacetate from L‐phenylalanine
Source: Microbiologyopen. 2017 Apr 24;6(4):e00486. doi: 10.1002/mbo3.486 (PMC5552962; doi:10.1002/mbo3.486)
Supplement: Supplementary file 1 [file MBO3-6-na-s001.doc]

Supporting Information for

**Metabolic Engineering of *Escherichia coli* for Production of**

**2-Phenylethylacetate from L-phenylalanine**

Daoyi Guo^1,2^*, Lihua Zhang^1,2^, Hong Pan^2^, Xun Li^2^

Corresponding author: Tel: +86-797-8353936; E-mail address: [ggdy3478@163.com](mailto:ggdy3478@163.com)

1. College of Life and Environmental Sciences, Gannan Normal University.

2. Key Laboratory of Organo-Pharmaceutical Chemistry, Jiangxi Province, Gannan Normal University, Ganzhou 341000, People’s Republic of China.


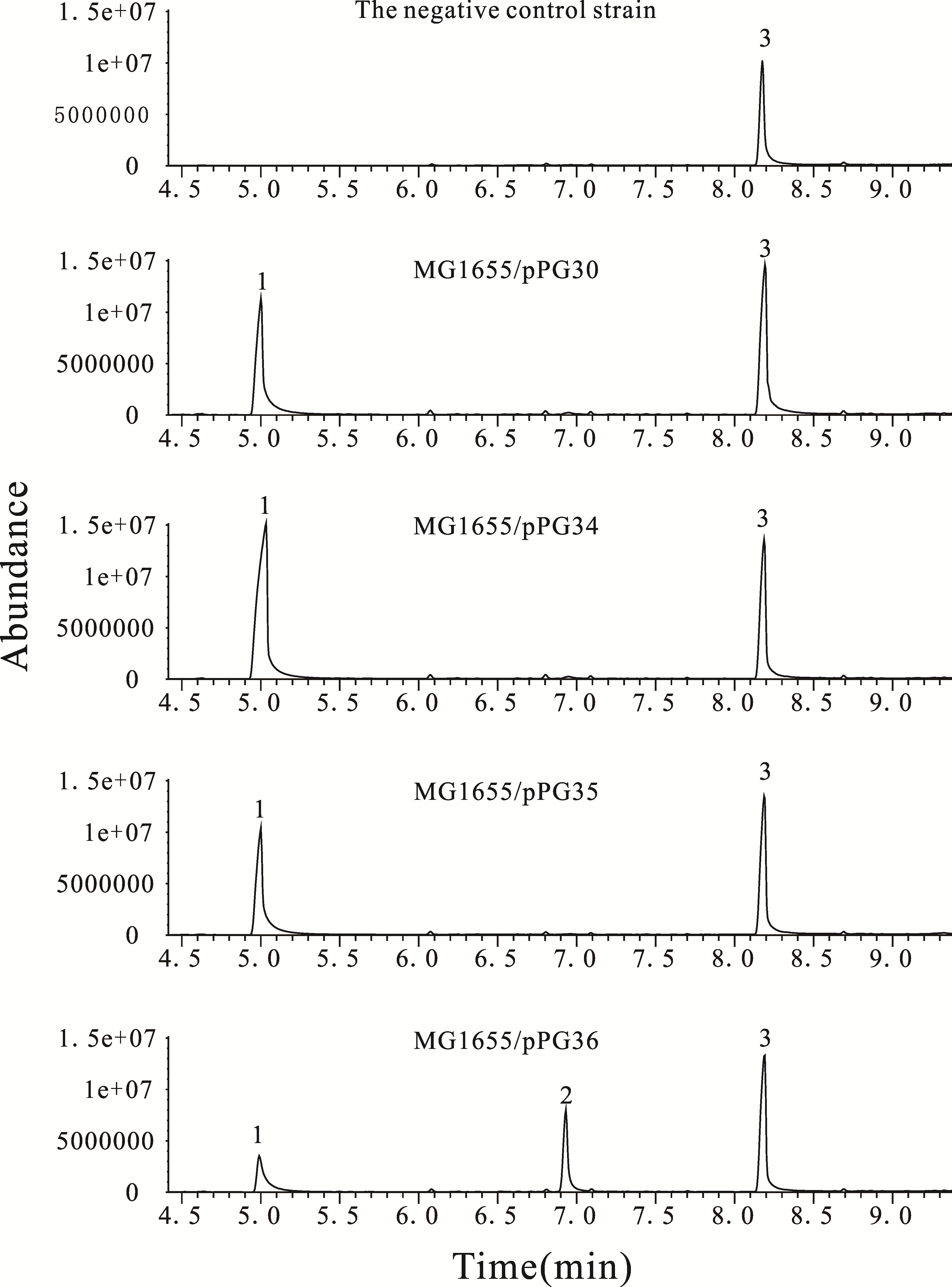


**Figure S1.** GC/MS analyses of 2-PE and 2-PEAc from glucose in engineered *E. coli* strains with modified M9 medium in shake flasks for 28 h. Identified substances: **1**, 2-PE; **2**, 2-PEAc; **3**, 2-Phenethylpropionat (internal standard).

**
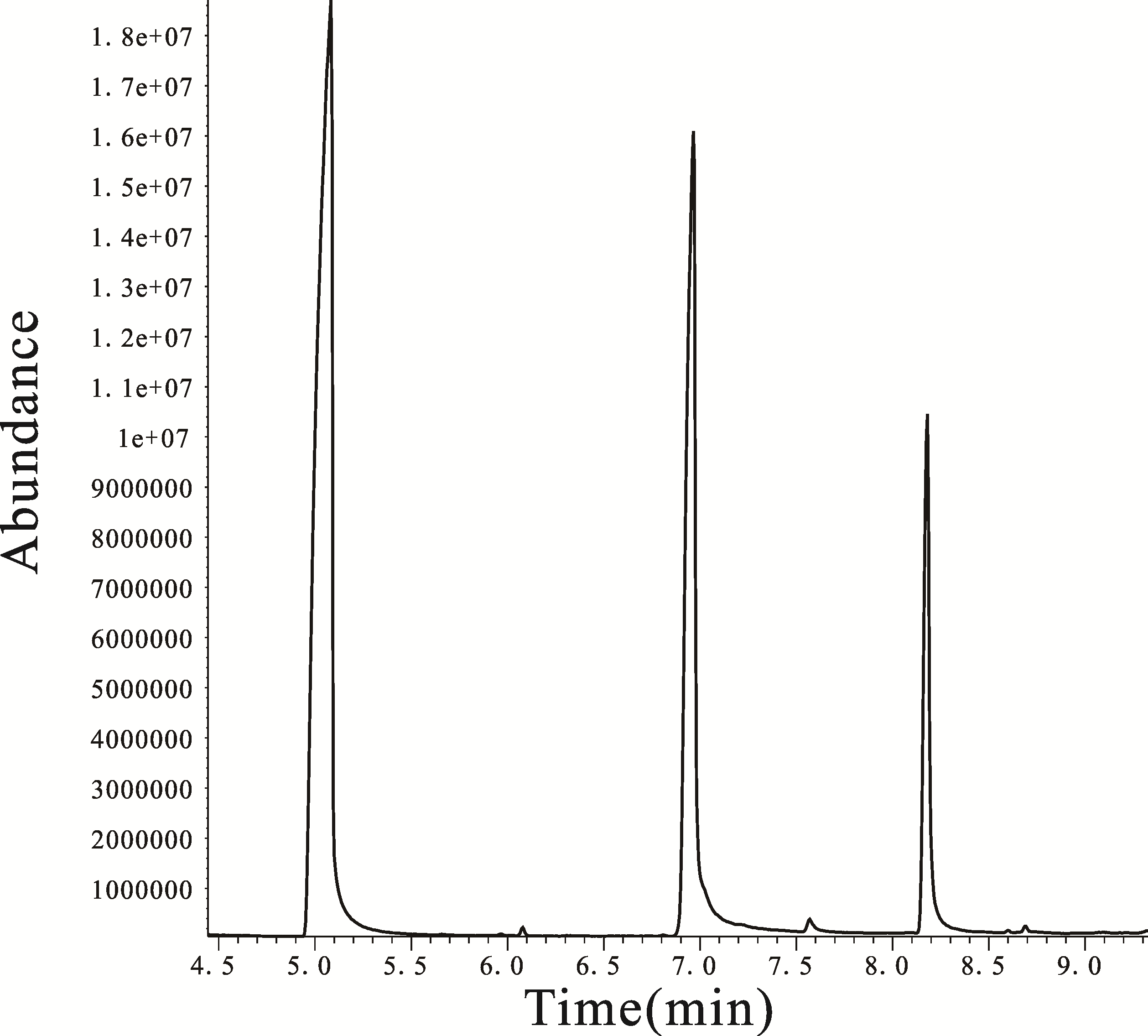
**

**Figure S2.** GC/MS analyses of 2-PE and 2-PEAc in engineered MG1655/pDG36 strains with modified M9 medium containing 1.0 g/L of phenylpyruvate in shake flasks for 28h. Identified substances: **1**, 2-PE; **2**, 2-PEAc; **3**, 2-Phenethylpropionat (internal standard).


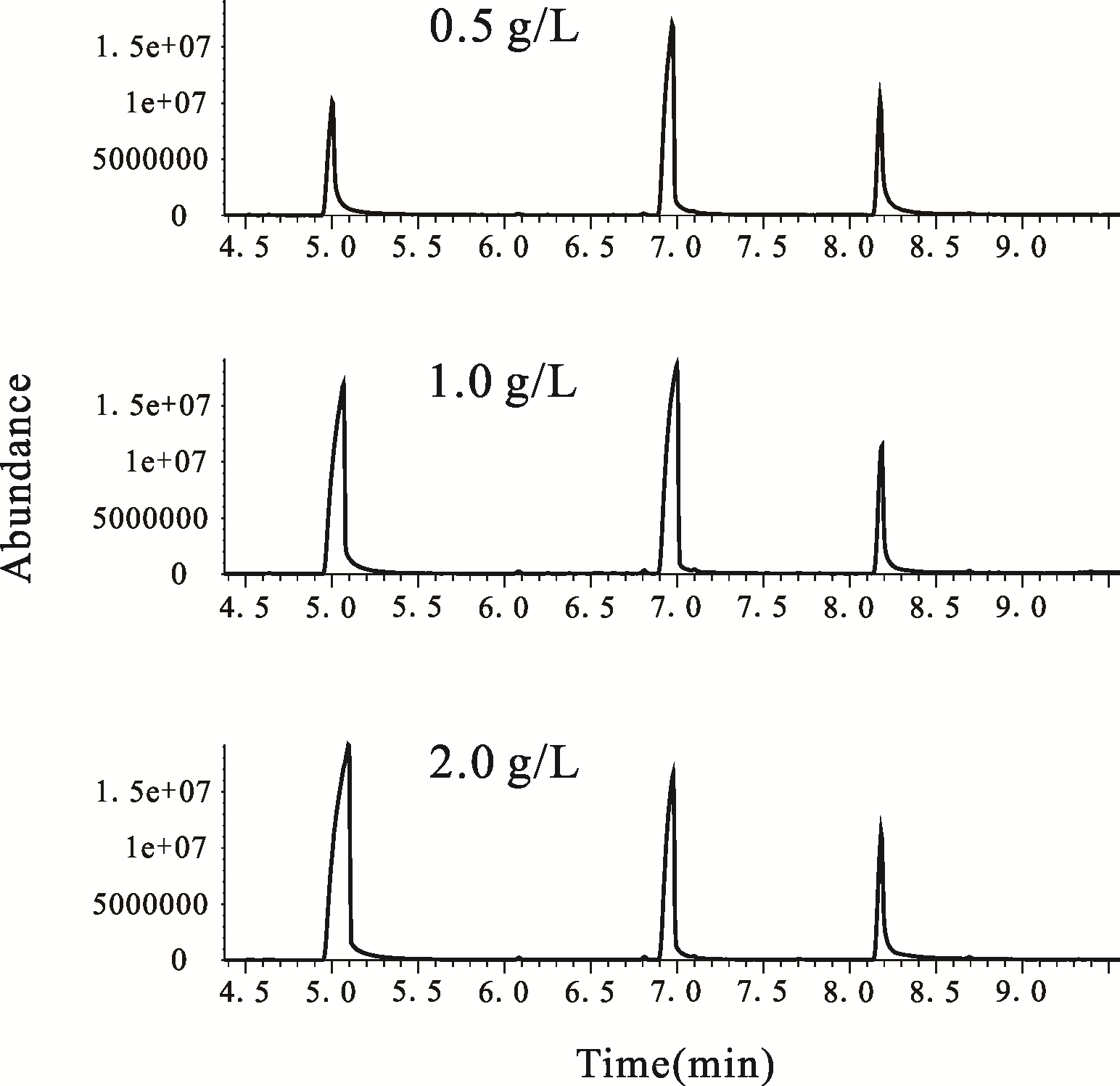


**Figure S3.** GC/MS analyses of 2-PE and 2-PEAc in engineered MG1655/pDG37 strains with modified M9 medium containing 0.5, 1.0, or 2.0 g/L of L-phenylalanine in shake flasks for 28h. Identified substances: **1**, 2-PE; **2**, 2-PEAc; **3**, 2-Phenethylpropionat (internal standard).
